# Plasma extrachromosomal circular DNA as a potential diagnostic biomarker for nodular thyroid disease

**DOI:** 10.1002/ctm2.1740

**Published:** 2024-06-20

**Authors:** Meng Zhou, Wei Lv, Peng Han, Kai Sun, Ziqian Hao, Ling Gao, Yunyun Xu, Zhe Xu, Shanshan Shao, Shizhan Ma, Qingling Guo, Haiqing Zhang, Ke Liu, Fan Yang, Zhongshang Yuan, Guojun Wu, Changbin Yu, Yonglun Luo, Zhenyu Yao, Jiajun Zhao

**Affiliations:** ^1^ Key Laboratory of Endocrine Glucose & Lipids Metabolism and Brain Aging, Ministry of Education, Department of Endocrinology Shandong Provincial Hospital Affiliated to Shandong First Medical University Jinan Shandong China; ^2^ Shandong Engineering Laboratory of Prevention and Control for Endocrine and Metabolic Diseases Jinan Shandong China; ^3^ Shandong Institute of Endocrine and Metabolic Disease Jinan Shandong China; ^4^ College of Life Sciences, University of Chinese Academy of Science Beijing China; ^5^ Lars Bolund Institute of Regenerative Medicine, HIM‐BGI Omics Center, Hangzhou Institute of Medicine (HIM), Chinese Academy of Sciences Hangzhou Zhejiang China; ^6^ College of Artificial Intelligence and Big Data for Medical Sciences, Shandong First Medical University & Shandong Academy of Medical Sciences Jinan Shandong China; ^7^ Shandong Provincial Hospital Affiliated to Shandong First Medical University Jinan Shandong China; ^8^ Central Hospital Affiliated to Shandong First Medical University Jinan Shandong China; ^9^ Department of Bio‐statistics School of Public Health Cheeloo College of Medicine, Shandong University Jinan Shandong China; ^10^ Department of Breast and Thyroid Surgery Shandong Provincial Hospital Affiliated to Shandong First Medical University Jinan Shandong China

Dear Editor,

The accurate diagnosis of cancerous nodules in nodular thyroid disease remains a significant challenge.^1^, ^2^ Extrachromosomal circular DNA (eccDNA), generated during apoptosis, shows tissue/plasma‐specific patterns, and varying spectra in different diseases. However, its role in distinguishing benign and malignant thyroid nodules is unexplored.^3^, ^4^, ^5^, ^6^, ^7^, ^8^, ^9^ This study leverages Circle‐seq technology and machine learning to investigate the potential of eccDNA as a non‐invasive biomarker for diagnosing thyroid cancer.

To understand the distribution of eccDNA in plasma amongst healthy controls (NOR, *N* = 13), nodular thyroid goitre patients (NOD, *N* = 25) and papillary thyroid carcinoma (PTC, *N* = 47) individuals, we applied an optimised Circle‐seq strategy for enriching circular DNA (Figure 1A), with the clinical characteristics of the subjects detailed in Table S1. The majority of the Circle‐seq reads were aligned to the human reference genome (Figure S1). We examined the eccDNA profiles in healthy controls and NOD/PTC patients, noting significant variability in eccDNA counts amongst groups. On average, NOR, NOD and PTC groups harboured 27 019 (range: 11 822–45 622), 16 850 (range: 7632–44 537) and 27 352 (range: 4015–83 301) unique eccDNAs, respectively. After normalising eccDNA counts to eccDNA counts per million mapped reads (EPM) to adjust for sequencing depth variations, the PTC group showed significantly elevated EPM values compared to both NOD and healthy controls (Figure 1B). Consistent with increased EPM values in PTC, this group also exhibited a higher number and proportion of eccDNA mapping to protein‐coding regions, while repeat element proportions were lower (Figure 1C–E). EccDNA load variations prompted us to explore its relationship with cancer characteristics, revealing that loss of eccDNA from coding regions correlates positively with tumour size, suggesting the specific roles of eccDNA in oncogenesis (Figure S2). The plasma eccDNA analysis in the PTC group showed longer fragments and higher Guanine‐Cytosine (GC) content, with most eccDNA populations under 1000 bases, peaking at around 202 and 338 bases, a preference for high GC content areas in eccDNA formation (Figure S3A–C). Genomic annotation highlighted eccDNA's enrichment in untranslated and exonic regions, aligning with known profiles in health and disease (Figure 1F). To understand the distribution of eccDNA genomic locations in PTC patients, we aligned eccDNA sequences with the human genome and identified 450 gene‐containing eccDNA (eccGenes) that were significantly prevalent in the PTC group (Figure S4A). Gene ontology (GO) and gene set enrichment analysis (GSEA) indicated that these eccGenes frequently involve exons related to tissue growth, and the Wnt and GPCR signalling pathways (Figure S4B,C). Principal component analysis (PCA) highlighted distinct eccDNA gene region diversity associated with PTC (Figure 1G). Remarkably, an increased presence of *miR‐1203*‐related eccDNA circles was detected in the PTC group (Figure 2A,B). Transfection of synthesised *miR‐1203* eccDNA into thyroid cell lines (TPC‐1, BHP10‐3 and K1) led to significant transcriptional changes: 572 genes were upregulated and 1035 downregulated (Figure 2C). This significant shift in gene expression, involving numerous cancer‐associated genes, underscores eccDNA's influence in oncogenesis (Figure 2D,E).

**FIGURE 1:**
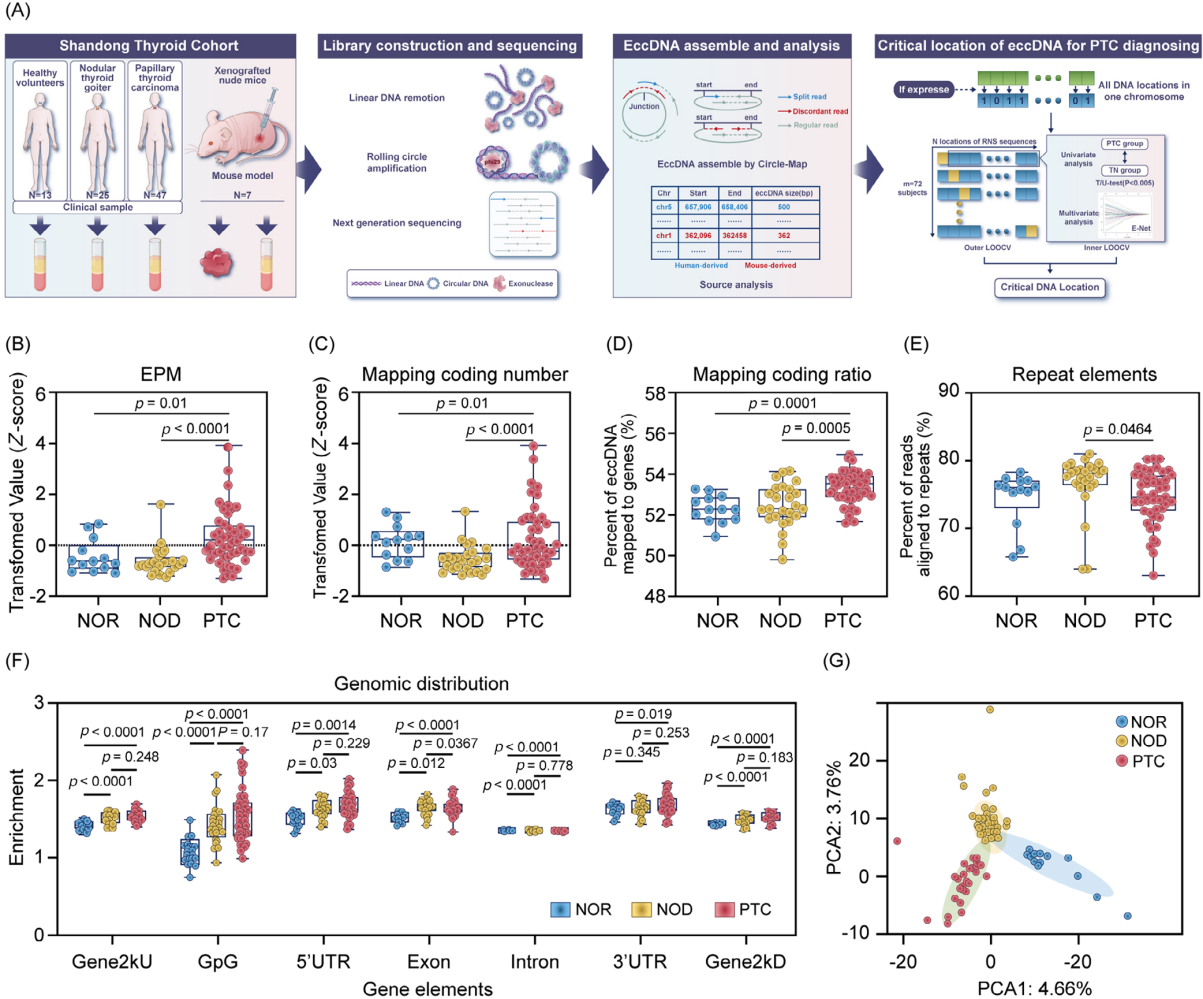
Distinct plasma extrachromosomal circular DNA (eccDNA) profiles in papillary thyroid carcinoma (PTC) patients. (A) Experimental workflow for identifying eccDNA from plasma and tumour tissues. DNA extraction and enrichment from tissue and plasma samples, followed by removal of linear DNA using exonuclease and amplification of eccDNA through rolling circle amplification. Machine learning analysis was conducted to pinpoint eccDNA locations capable of differentiating PTC and nodular thyroid goitre (NOD) patients. (B–G) Genomic and sequence characteristics of plasma eccDNA. (B–E) The eccDNA counts per million mapped reads (EPM) value, number of eccDNA mapped to protein‐coding genes, percent of eccDNA mapped to protein‐coding genes and percent of reads aligned to repeats in each clinical group. (F) Genomic distribution of eccDNA in each clinical group. (G) Principal component analysis (PCA) of differential eccGenes in each clinical group. *p* values are shown on the graph.

**FIGURE 2:**
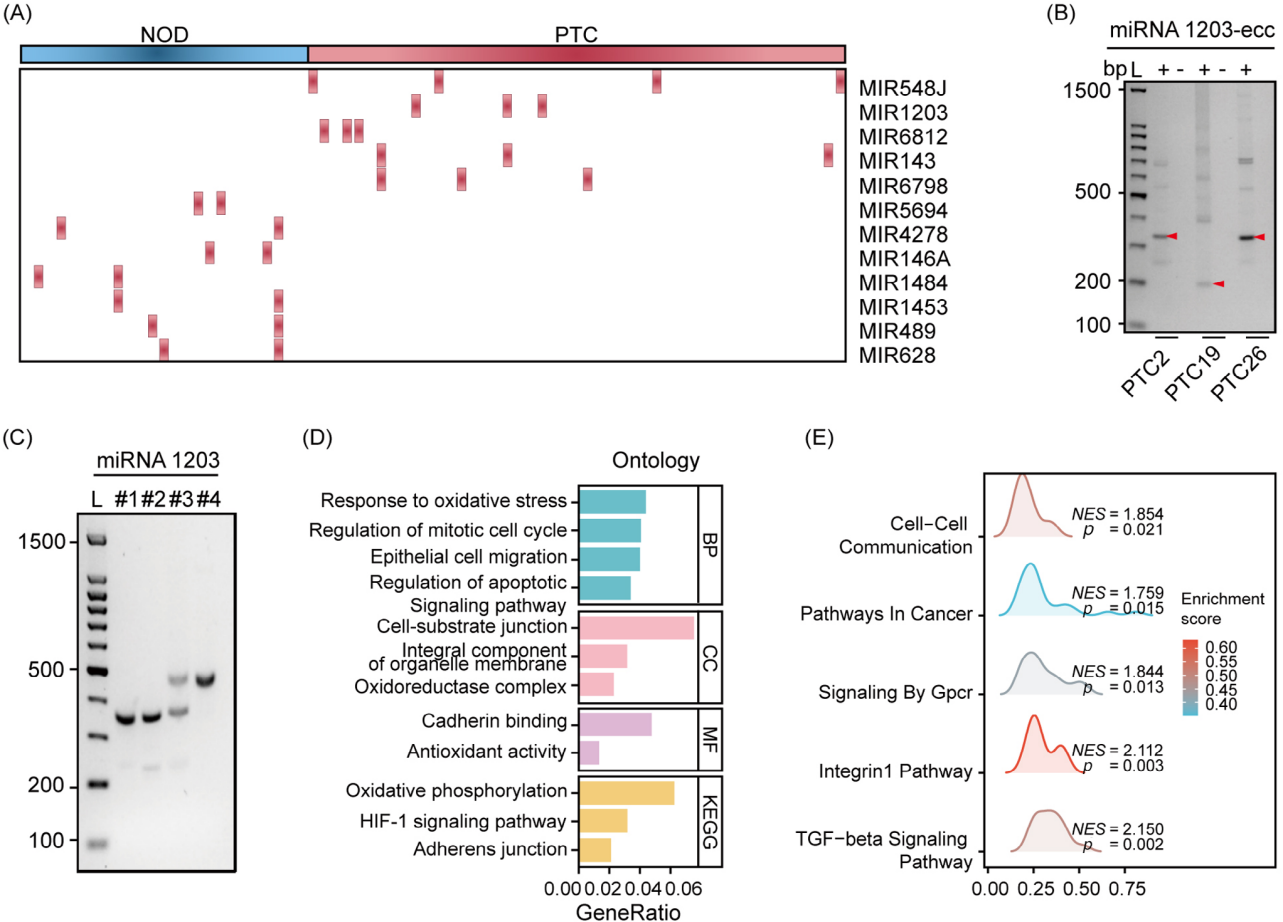
*miR‐1203*‐related circles promote the progression of papillary thyroid carcinoma (PTC). (A) Heatmap showing extrachromosomal circular DNA (eccDNA) containing full‐length miRNA genes abundance between the PTC and nodular thyroid goitre (NOD) groups. (B) Validation of miRNA1203‐related eccDNAs by agarose gel electrophoresis. (C) Synthesis of miRNA1203‐eccDNAs by the Ligase‐Assisted Mini‐circle Accumulation (LAMA) method. (D, E) Gene ontology (GO), KEGG and gene set enrichment analysis (GSEA) pathway analysis of miRNA1203‐eccDNA biological functions.

To investigate if PTC tumour cells emit eccDNA into the bloodstream, we crafted mouse xenograft models with human PTC cell lines TPC‐1, BHP10‐3 and K1. We isolated eccDNA from plasma, confirming human‐origin eccDNA in mice (Figure 3A). Sequencing revealed genomic features consistent with tumour‐derived eccDNA, including GC content, motif patterns and chromosomal distribution, highlighting its potential as a cancer detection marker (Figure 3B–D).

**FIGURE 3:**
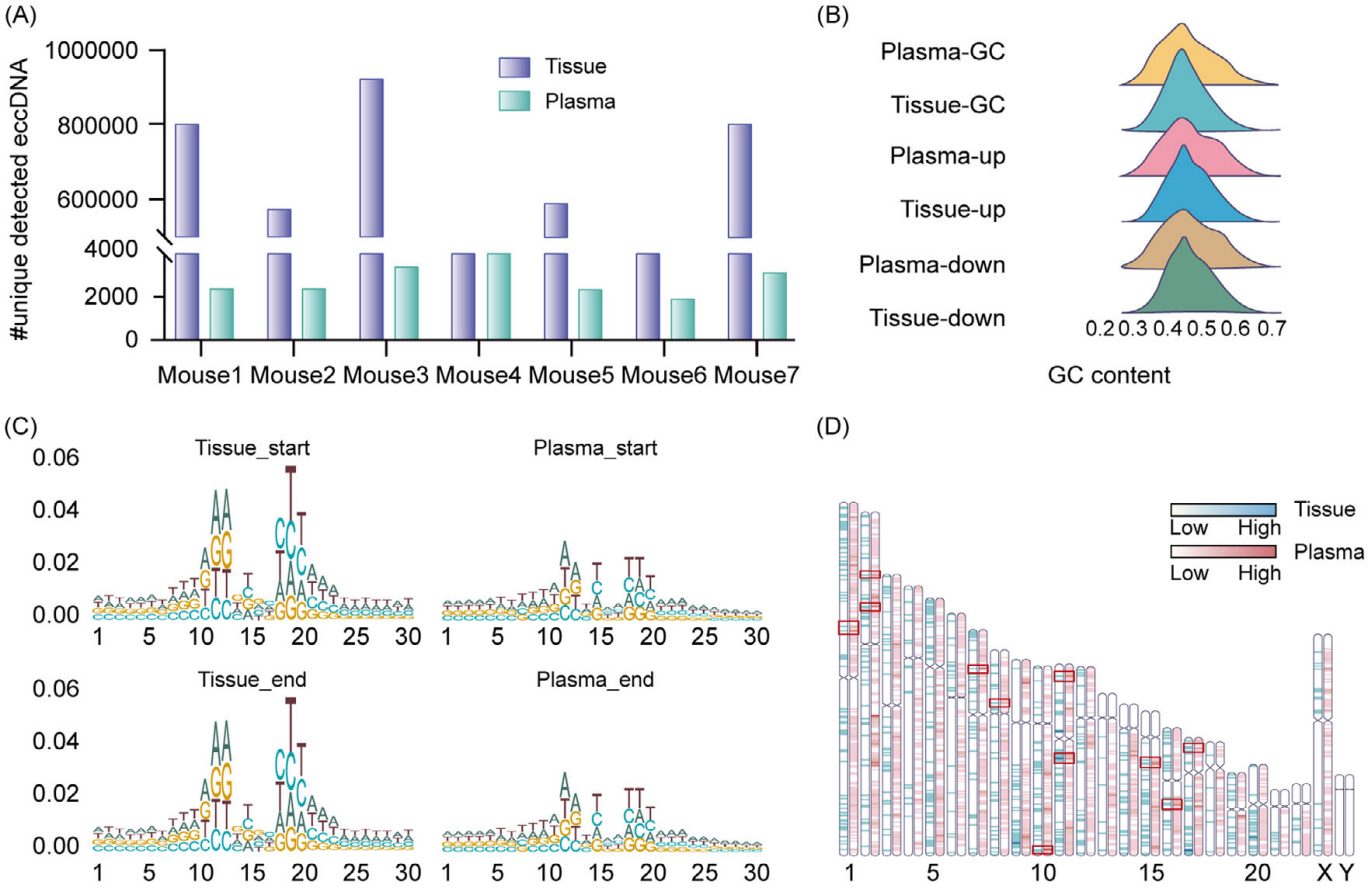
Characteristics of human extrachromosomal circular DNA (eccDNA) in tissue and plasma of human papillary thyroid carcinoma (PTC) cell xenografts mice. (A) Summary of the number of eccDNA of mouse or human origins detected in xenograft mouse plasma. (B) GC content distribution of eccDNA and their up‐ and down‐stream regions with equal length in tissue and plasma. (C, D) Motif patterns of eccDNA junction sites and chromosomes distribution of PTC cell line xenograft mouse tissue and plasma.

Exploring eccDNA's diagnostic value in PTC, we analysed 308 837 genomic locations. Utilising an E‐net logistic regression model and a two‐nested leave‐one‐out strategy,^10^ we assessed the link between these locations and disease status. We identified 71 critical eccDNA locations capable of differentiating PTC from NOD patients, forming a potential classification model. By comparing 71 eccDNA locations identified in this study with the gene annotations of the human genome GRCh38.p13 in Ensembl (version 108), we found 18 locations showed no overlap with any known genes, while the remaining 53 locations overlapped with 71 known genes (Figure 4A and Table S2).

**FIGURE 4:**
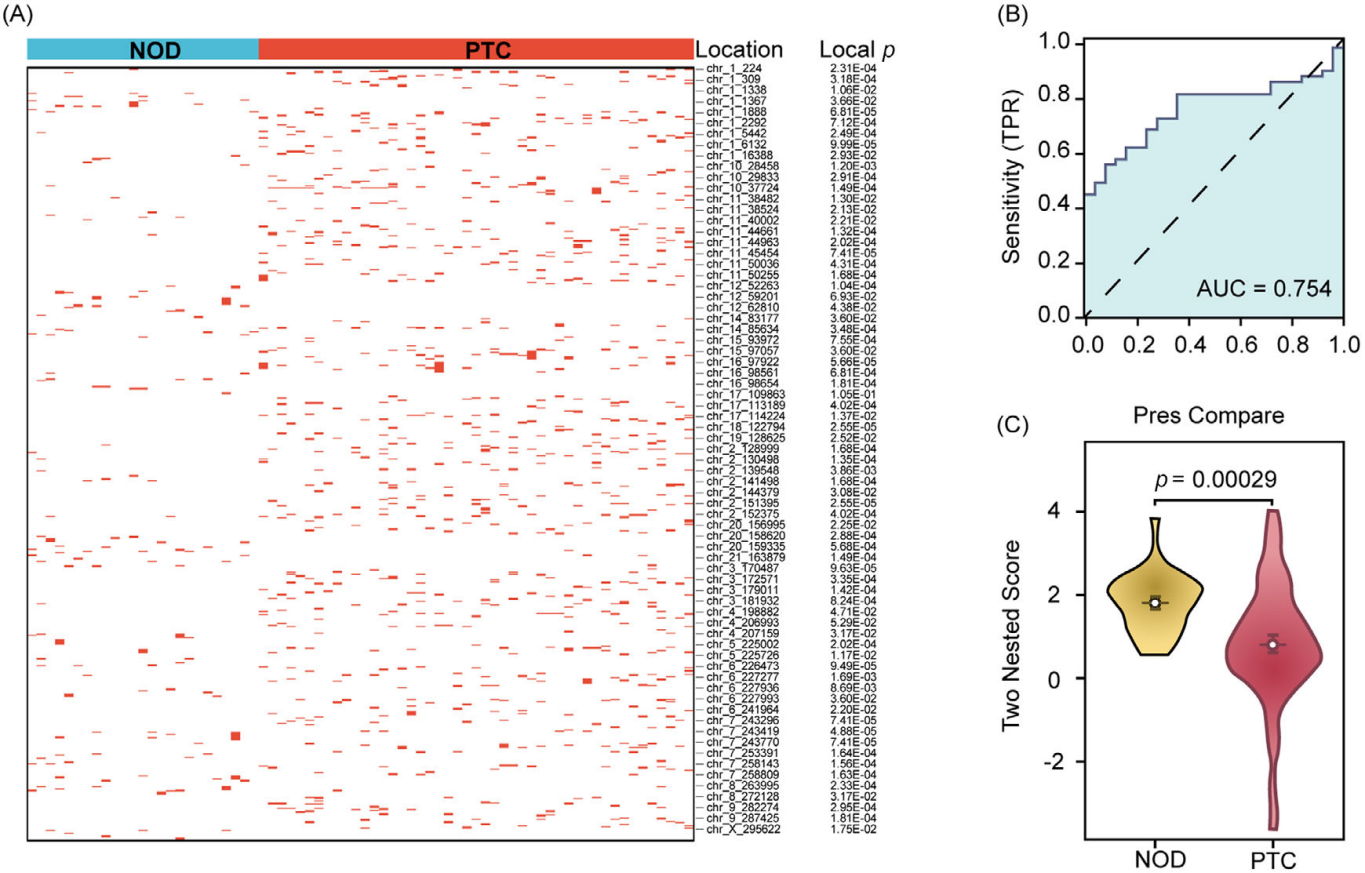
Evaluation of the critical locations of extrachromosomal circular DNA (eccDNA) for papillary thyroid carcinoma (PTC) diagnosing. (A) Summary of the 71 crucial locations for classification model capable of distinguishing the PTC and nodular thyroid goitre (NOD) patients. (B) Area under the curve (AUC) value by Receiver Operating Characteristic (ROC) analysis using the 72 predictions and their corresponding true labels. (C) Boxplot of the 72 two‐nested predicted scores calculated by the 71 locations for PTC and NOD patients. *p* values are shown on the graph.

After completing the two‐nested leave‐one‐out cross‐validation (LOOCV) process, we generated 72 predictions for the 72 subjects. Receiver operating characteristic curve (ROC) analysis on these predictions and their true labels produced an area under the curve (AUC) of .754 (Figure 4B). To further evaluate the diagnostic efficacy of our model, we have supplemented it with more detailed performance metrics, including an accuracy of 68.1%, sensitivity of 55.3%, specificity of 92.0%, positive predictive value of 92.9% and negative predictive value of 52.3%. We also selected the optimal cut‐off point using a two‐nested LOOCV approach. To address possible bias from random training and validation set splits, we conducted 2000 rounds of five‐fold cross validation, averaging an AUC of .789 with a standard deviation of .135. Additionally, a *T*‐test comparing predictions across the two groups indicated a statistically significant difference (*T* = −3.9012, *p* = .00029; Figure 4C), suggesting the 71 identified eccDNA locations could serve as a diagnostic biomarker for distinguishing between PTC and NOD patients.

This study explores the potential of plasma eccDNA as a non‐invasive biomarker to distinguish between benign and malignant thyroid nodules, potentially improving the diagnosis of PTC. The elevated eccDNA levels found in PTC patients and mouse PTC xenograft models indicate its tumour‐originated release into circulation, supporting its diagnostic value. In this study, the sample size is an important limitation, which restricts the exploration of research conclusions and the performance of the model to a certain extent. A small sample size may lead to overfitting and reduce the credibility of research conclusions. To address this, we adopted a two‐nested LOOCV loops to ensure that the testing set in the outer loop does not involve repeated training. The results from the outer loop were used as evaluation criteria, maximising data utilisation and preventing overfitting. After our posterior sample size estimation, our sample size meets the minimum sample size requirement, indicating that the research conclusions are credible. In the future, we will expand the sample size to improve our research model and conclusions.

Despite limited samples, we identified the nucleosomal origin and genetic predisposition for eccDNA formation in PTC, establishing eccDNA as a promising biomarker for cancer detection and enhancing thyroid cancer diagnostics precision.

## AUTHOR CONTRIBUTIONS

Jiajun Zhao, Yonglun Luo, Ling Gao and Changbin Yu conceived the idea. Meng Zhou, Zhenyu Yao and Yunyun Xu collected the clinical samples and constructed the mouse xenograft models. Wei Lv and Peng Han performed the Circle‐seq experiments. Yonglun Luo, Wei Lv, Meng Zhou, Peng Han, Kai Sun and Ziqian Hao drafted most of the manuscript. Wei Lv, Peng Han and Zhe Xu analysed the eccDNA data. Kai Sun, Ziqian Hao and Meng Zhou encoded the eccDNA sequences and performed machine learning and statistical analysis. Shanshan Shao, Shizhan Ma, Qingling Guo, Haiqing Zhang, Ke Liu, Fan Yang, Zhongshang Yuan, Jiajun Zhao and Guojun Wu have contributed to the execution of the experiments and studies. Meng Zhou, Wei Lv, Peng Han, Kai Sun, Ziqian Hao, Guojun Wu, Changbin Yu, Zhenyu Yao, Ling Gao and Jiajun Zhao discussed the results and contributed to the final manuscript.

## CONFLICT OF INTEREST STATEMENT

The authors declare no conflicts of interest.

## Supporting information

Supporting Information

## References

[1] Xu H , Zhang Y , Wu H , et al. High diagnostic accuracy of epigenetic imprinting biomarkers in thyroid nodules. J Clin Oncol. 2023;41(6):1296‐1306. 36378996 10.1200/JCO.22.00232 PMC9937101

[2] Li X , Zhang S , Zhang Q , et al. Diagnosis of thyroid cancer using deep convolutional neural network models applied to sonographic images: a retrospective, multicohort, diagnostic study. Lancet Oncol. 2019;20(2):193‐201. 30583848 10.1016/S1470-2045(18)30762-9 PMC7083202

[3] Noer JB , Horsdal OK , Xiang X , Luo Y , Regenberg B . Extrachromosomal circular DNA in cancer: history, current knowledge, and methods. Trends Genet. 2022;38(7):766‐781. 35277298 10.1016/j.tig.2022.02.007

[4] Moller HD , Mohiyuddin M , Prada‐Luengo I , et al. Circular DNA elements of chromosomal origin are common in healthy human somatic tissue. Nat Commun. 2018;9(1):1069. 29540679 10.1038/s41467-018-03369-8 PMC5852086

[5] Hung KL , Yost KE , Xie L , et al. ecDNA hubs drive cooperative intermolecular oncogene expression. Nature. 2021;600(7890):731‐736. 34819668 10.1038/s41586-021-04116-8 PMC9126690

[6] Sin STK , Jiang P , Deng J , et al. Identification and characterization of extrachromosomal circular DNA in maternal plasma. Proc Natl Acad Sci U S A. 2020;117(3):1658‐1665. 31900366 10.1073/pnas.1914949117 PMC6983429

[7] Lv W , Pan X , Han P , et al. Circle‐Seq reveals genomic and disease‐specific hallmarks in urinary cell‐free extrachromosomal circular DNAs. Clin Transl Med. 2022;12(4):e817. 35474296 10.1002/ctm2.817 PMC9042798

[8] Zou S , Chen S , Rao G , et al. Extrachromosomal circular MiR‐17‐92 amplicon promotes HCC. Hepatology. 2024;79(1):79‐95. 37125628 10.1097/HEP.0000000000000435

[9] Wang Y , Wang M , Djekidel MN , et al. eccDNAs are apoptotic products with high innate immunostimulatory activity. Nature. 2021;599(7884):308‐314. 34671165 10.1038/s41586-021-04009-w PMC9295135

[10] Tirumalaraju V , Suchting R , Evans J , et al. Risk of depression in the adolescent and adult offspring of mothers with perinatal depression: a systematic review and meta‐analysis. JAMA Netw Open. 2020;3(6):e208783. 32602910 10.1001/jamanetworkopen.2020.8783 PMC7327545

