# Supplementary material for: Plasma extrachromosomal circular DNA as a potential diagnostic biomarker for nodular thyroid disease
Source: Clin Transl Med. 2024 Jun 20;14(6):e1740. doi: 10.1002/ctm2.1740 (PMC11189768; doi:10.1002/ctm2.1740)
Supplement: Supplementary file 1 — Supporting Information [file CTM2-14-e1740-s001.docx]

**Supplemental Materials**

**This PDF file includes:**

**Materials and Methods**

**Availability of Data and Materials**

**Supplementary Figure 1-4**

**Supplementary Table 1-4**

**List of abbreviations**

**Materials and Methods**

**Study design, case recruitment, and sample processing**

The main objective of this study was to examine the plasma eccDNA patterns in individuals with papillary thyroid cancer (PTC) and non-neoplastic thyroid nodules (NOD) and to investigate the potential clinical implications of eccDNA. Ethical approval for this study was obtained from the Medical Ethics Committee of the First Affiliated Hospital of Shandong First Medical University. We adhered to the Transparent Reporting of a multivariable prediction model for Individual Prognosis or Diagnosis (TRIPOD) statement, thereby enhancing the reliability of the guideline and feature selection and review process.^1,2^ A total of 47 patients diagnosed with PTC, 25 patients diagnosed with NOD, and 13 normal thyroid volunteers (NOR) participated in this study. All participants provided written informed consent. The patients with thyroid cancer and thyroid nodules were pathologically confirmed through total thyroidectomy. The basic clinical features of these patients are listed in **Table S1**. Plasma samples were separated from peripheral blood and centrifuged at 16000 *g* for 10 min at 4°C and were stored at -80°C for further process.

**Purification of plasma/tissue eccDNA**

EccDNA was purified from plasma and tissues using the Circle-Seq method as we have previously described.^3,4^ *For plasma cfDNA isolation*: About 500 µL of plasma samples in 1.5 mL Eppendorf tubes added with 20 µl proteinase K were incubated at 55°C at 600 rpm vortex (Eppendorf Thermomixer) for 20 min. Then, total cfDNAs (consisting of both linear DNAs and circular DNAs) were extracted from plasma using an MGIEasy Circulating DNA Extraction Kit (MGI-BGI, China) according to the manufacturer’s protocol. cfDNA was eluted in 45 µL RNase-free water and 1 µL of cfDNA was taken for concentration analysis by Qubit Hs DNA dsDNA High Sensitivity assay on Qubit 3.0 Fluorometer (Invitrogen). *Removal of cell-free linear DNA:* To remove the linear portions of cfDNA and enrich for circular DNA (consisting of both mitochondrial DNA and eccDNA), 40 µL of cfDNA (5- 50 ng) was digested with the 20 units of Plasmid-Safe DNase (PSD, 10,000u/ml, Epicenter) at 37°C for 16 h in a 50 µL reaction system. The digestion products were recovered with 90 µL WAHTS® DNA clean Beads (Vazyme) and eluted in 24 µL RNase-free water. *Rolling circle amplification (RCA):* To increase the signal, the number of enriched traces of circular DNA (12 µL out of a total of 24 µL) was amplified greatly via RCA and the RCA reaction system involved. *For tissues*: Total high molecular weight (HMW) DNA was extracted from mouse tumor tissues and cell lines using MagAttract HMW DNA kit (Qiagen), according to the manufacturer’s instructions. Then the linear portions of HMW DNA were treated with 20U PSD at 37°C for 72h. The clean and RCA steps followed the protocol of plasma eccDNA purification. To avoid possible effects of batch-to-batch variation, all clinical samples were processed by the same operator (W.L.).

**Library preparation and eccDNA deep sequencing**

The φ29-amplified DNA samples (0.5 µg) were firstly sonicated into a 300-500 bp size range on the Covaris LE220 (Covaris). Then, 80 ng DNA fragments were end-repaired, A-tailed, and adapter-ligated using the MGIEasy DNA Library Preparation Kit (MGI-BGI, China). The quality control (including size distribution and concentration) of each library was assessed by the Agilent Bioanalyzer 2100 system. Lastly, the constructed library was deep sequenced on the MGI PE150 platform (BGI, China).

**EccDNA assembly by Circle-Map**

Sequencing reads were mapped to the human/ mouse reference genome (ref. hg38/mm10) using BWA.^5^ Circle-Map (V1.1.4) software was adapted to call circular DNA from Circle-Seq data based on junction reads. The filtration parameter was performed as we previously described.^3^

**EccDNA abundance analysis**

To explore the eccDNA patterns among different clinical groups. We used the eccDNA abundance for statistics and the eccDNA abundance was calculated as the number of eccDNA on a specific gene divided by the gene length and total detected circles.

**Gene pathway analysis**

Gene ontology and KEGG pathway analysis were performed by ClusterProfiler V4.4.4.^6^

**EccDNA validation by PCR**

eccDNA validation was performed by outward PCR and the PCR primers were listed in **Table S3**. Each 30 μL PCR reaction system included 50 μg phi29-amplified DNA products, 500 nM primer, 15 μL NEBNext High-Fidelity 2X PCR Master Mix (NEB), and PCR reaction for 40 cycles. All reactions were performed accompanied by non-template control (NTC). The PCR products were tested by agarose (2%) gel electrophoresis, and the target products were recovered by QIAEX II Gel Extraction Kit.

**Synthesis of artificial eccDNA**

The artificial eccDNA were synthesized by the ligase-assisted minicircle accumulation (LAMA) system as previously described^7^ and LAMA primers of MRI1203 eccDNA were listed in **Table S4**.

**Cell culture and transfection (Transfection efficiency evaluation)**

The human thyroid cell lines TPC-1, BHP10-3, and K1 were obtained from American type culture collection (ATCC) or the cell bank in the Chinese Academy of Sciences (Shanghai, China). All cell lines were stored in Shandong Provincial Hospital and maintained in RPMI-1640 (GIBCO, USA) or DMEM (GIBCO, USA) supplemented with 10% (v/v) fetal bovine serum (FBS) (GIBCO, USA). Cells were incubated at 37 °C in a humidified atmosphere with 5% CO_2_ (5 L CO_2_/95 L atmospheres). Thyroid cells were seeded at 3×10^5^ per well in the 6-well plates and allowed to attach for at least 12 h. To assess the effects of eccDNA on thyroid cell tumor activity, 100 ng eccDNA or random eccDNA control was transfected into the cells using the Lipofectamine 3000 (ThermoFisher, USA) according to the manufacturer’s instruction. At 4 h post-transfection, culture medium was replaced with medium containing 10% FBS, and 48 h after transfection, RNA was extracted for transcriptome detection.

**Differential gene expression (DGEs)**

Differential expression analysis of two groups (artificial circles transfected with random sequence control and miRNA-1203) was performed using the DESeq R package (1.10.1). Genes with an adjusted *P* value < 0.05 found by DESeq were assigned as differentially expressed.

**Construction of mouse xenograft models**

All xenograft experiments were conducted with the approval and supervision of the Animal Care & Welfare Committee of Shandong Provincial Hospital. BALB/c nude mice were obtained from Charles River Laboratories and maintained in the Animal Center of Shandong Provincial Hospital under specific pathogen-free (SPF) conditions. The mice were cared for according to Chinese animal welfare legislation and under the NIH Guidelines of Care and Use of Laboratory Animals. For the xenografted tumor model, 5 × 10^6^ cells of each thyroid cancer cell line (i.e., TPC-1, BHP10-3 and K1) were suspended in 100 μL medium and subcutaneously injected into the nude mice. Seven weeks after the inoculation of tumor cells, a sample of approximately 500 μL of blood was extracted from the inner canthal orbital vein and subjected to centrifugation to obtain the supernatant. Subsequently, the mice were subjected to cervical dislocation, and the tumor tissue was promptly frozen for Circle-Seq analysis.

**Encoding the eccDNA**

Based on our prior findings, the majority of eccDNA exhibited a length of less than 1kb. Consequently, the genome was partitioned into 10 kb windows, which were subsequently subdivided into 10 smaller 1 kb fragments. For each subject, the number of overlaps between all eccDNA fragments and each window was calculated. In the event that a subject's eccDNA did not overlap with any small fragments within a given window, the code for that subject within that window would be 0. If that the eccDNA of an individual exhibited concurrence with all 10 discrete small fragments within a specified region, the corresponding code for the said individual within the corresponding region would be 10. Subsequently, all individuals were assigned codes and a matrix was generated, encompassing all individuals and their respective genomic positions.

**Selection of critical location of eccDNA**

Following the encoding process, the location of the eccDNA sequence was transformed into a quantitative variable and subsequently utilized as the independent variable for subsequent analysis. The two-nested leave-one-out cross-validation (LOOCV) strategy was employed to identify the most significant location features.

Specifically, the present study employed a two-nested LOOCV approach, comprising an outer LOOCV loop and an inner LOOCV loop. In the outer LOOCV loop, one subject was left out as the testing dataset, and the rest subjects in the current loop were used as the training dataset. All subjects were left out, in turn, as the test dataset. In the training dataset, a univariate analysis was used to select the discriminative variables between the PTC group and NOD group first, and the variables for which *P*-values were smaller than 0.005 were retained. The univariate analysis employed either the two-sided Student's T-test or the Wilcox U-test, contingent upon the variable's adherence to normal distribution. Subsequently, an E-net logistic model was constructed, utilizing the features retained from the univariate analysis as independent variables and the label (PTC or NOD) as the dependent variable. The Alpha parameter of the E-net logistic model was established at 0.1, while the optimal Lambda parameter was determined through the inner LOOCV loop. The parameter selection and model-building process were performed using R 4.2.1 (Glmnet package v 4.1-4). The aforementioned procedure was iterated 72 times, corresponding to the number of subjects, whereby each subject was excluded as the testing dataset in turn in the outer LOOCV loop. Following the completion of the two-nested LOOCV loop, 72 distinct E-net logistic models were generated. The variables that were consistently retained across all E-net logistic models were identified as the most crucial locations and were subsequently employed in the ensuing analysis.

**Evaluation of the diagnostic potential of selected critical eccDNA**

Following the two-nested LOOCV loops, 72 predictions of these subjects were calculated by the 72 E-net logistic models. The discriminatory potential of the selected critical locations of eccDNA in distinguishing between PTC and NOD patients was assessed using ROC analysis. Additionally, a ridge regression model was constructed with a five-fold cross-validation strategy, which 80% of the subjects (four folds) randomly selected from cross-validation, and the model's performance was evaluated on the remaining 20% of the samples (one-fold). In order to mitigate the effects of randomization resulting from the division of the training and validation cohorts, we conducted 2000 iterations of cross-validation to estimate the classification performance. The classification performance of the selected features was assessed by calculating the mean AUC values across these 2000 iterations.

**Statistical Analysis**

All statistical tests and machine learning analysis were implemented by the R-4.1.1. E-net model built by the package Glmnet. The difference comparison of the two groups was performed by Student’s T-test or the Wilcox U-test according to whether the variable satisfies the normal distribution.

**Ethics declarations**

Ethical approval for human and animal experiments was obtained from the Medical Ethics Committee of the First Affiliated Hospital of Shandong First Medical University.

**Reference**

1. Collins GS, Reitsma JB, Altman DG, Moons KG. Transparent reporting of a multivariable prediction model for individual prognosis or diagnosis (TRIPOD): the TRIPOD statement. BMJ 2015; 350: g7594.

2. Wu WT, Li YJ, Feng AZ, et al. Data mining in clinical big data: the frequently used databases, steps, and methodological models. Mil Med Res 2021; 8(1): 44.

3. Lv W, Pan X, Han P, et al. Circle-Seq reveals genomic and disease-specific hallmarks in urinary cell-free extrachromosomal circular DNAs. Clin Transl Med 2022; 12(4): e817.

4. Xu Z, He J, Han P, et al. Plasma extrachromosomal circular DNA is a pathophysiological hallmark of short-term intensive insulin therapy for type 2 diabetes. Clin Transl Med 2023; 13(10): e1437.

5. Md V, Misra S, Li H, Aluru S. Efficient Architecture-Aware Acceleration of BWA-MEM for Multicore Systems. 2019 IEEE International Parallel and Distributed Processing Symposium (IPDPS); 2019; 2019.

6. Wu T, Hu E, Xu S, et al. clusterProfiler 4.0: A universal enrichment tool for interpreting omics data. Innovation (Camb) 2021; 2(3): 100141.

7. Du Q, Kotlyar A, Vologodskii A. Kinking the double helix by bending deformation. Nucleic Acids Res 2008; 36(4): 1120-8.

**Availability of Data and Materials**

The sequencing data has been deposited in GSA-Human (https://ngdc.cncb.ac.cn/) under the accession number HRA004809.

**Supplementary Figures**


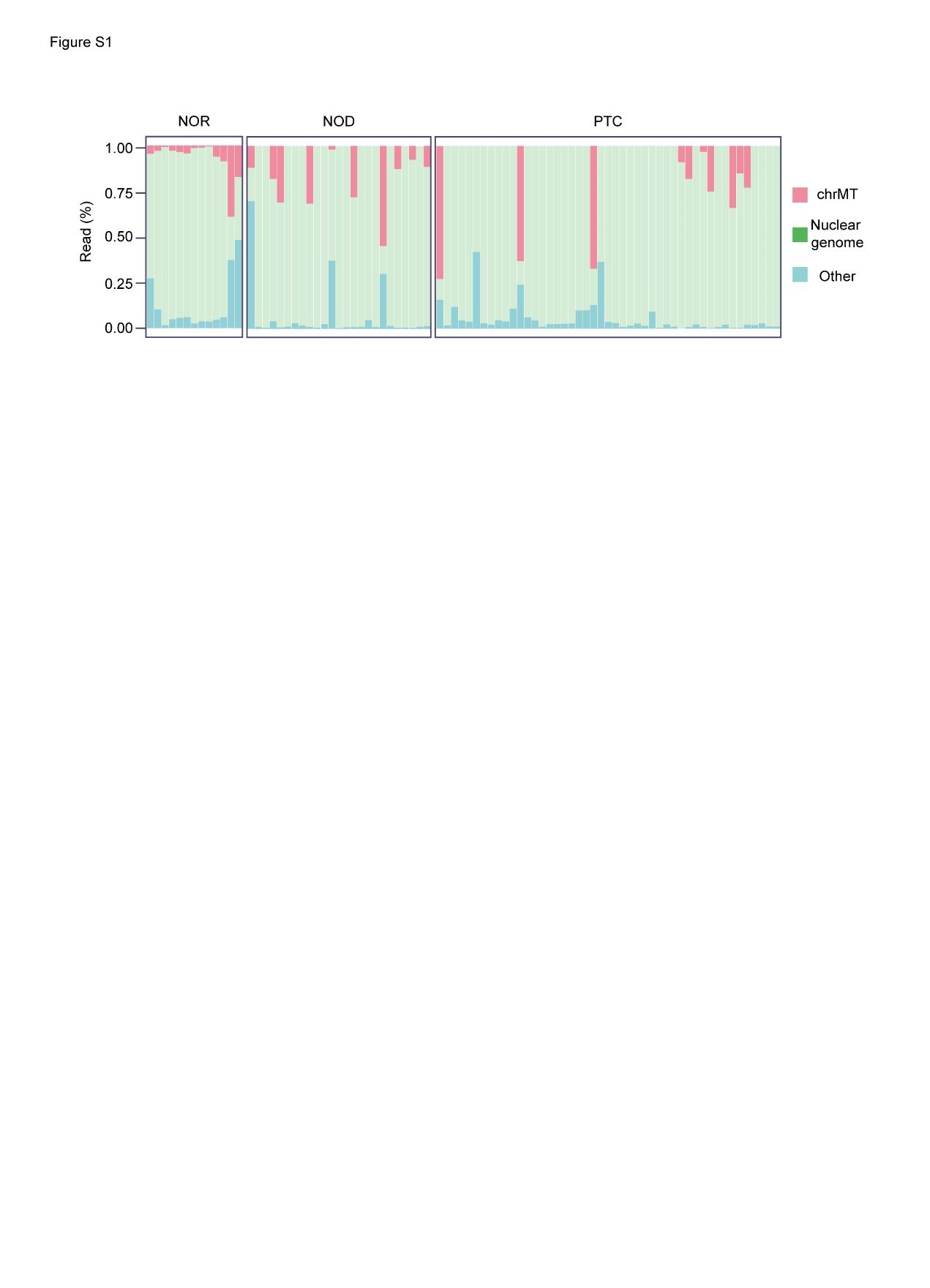


Figure S1. Mapping results of Circle-seq reads. MT, mitochondrial.


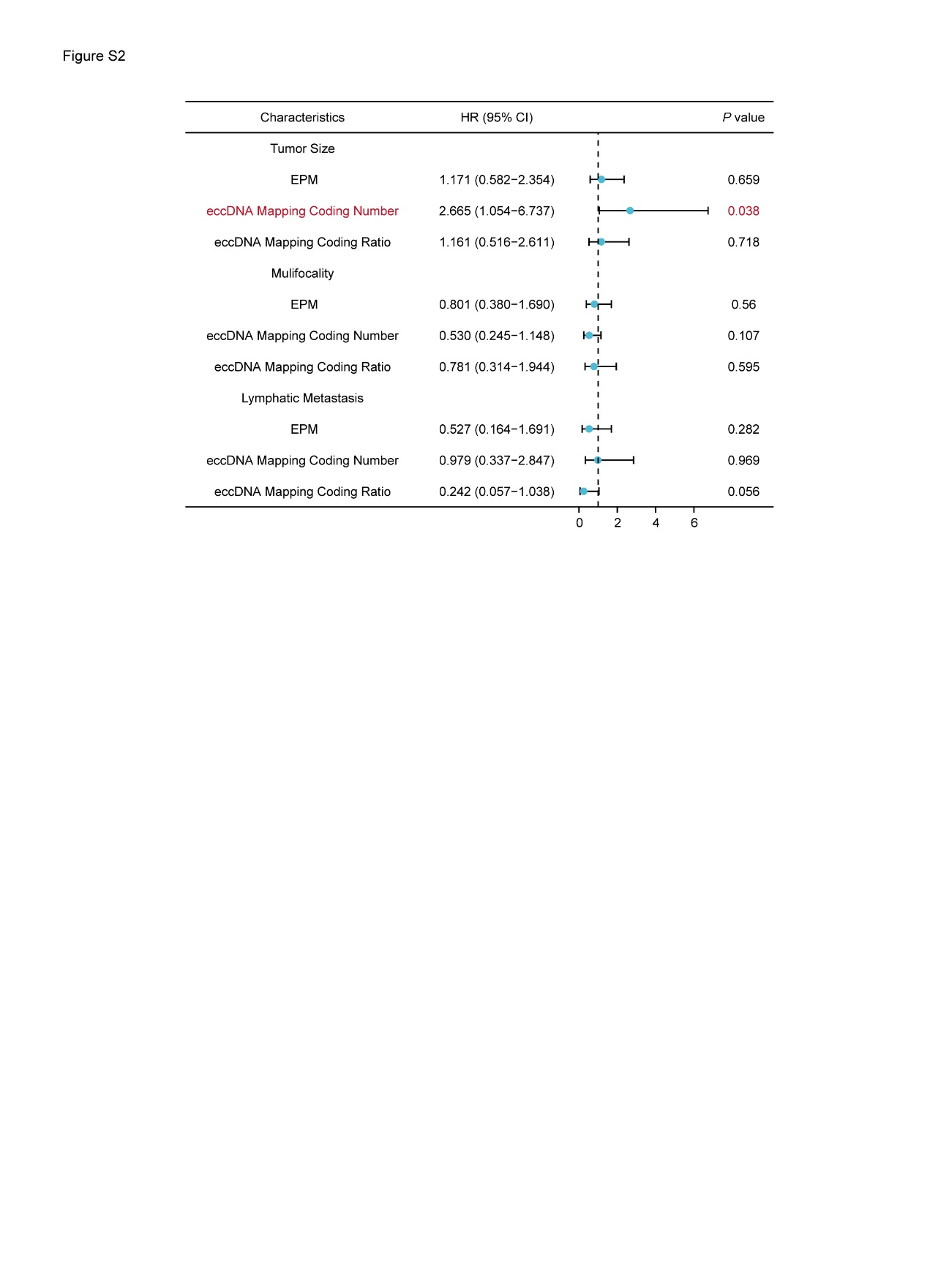


Figure S2. Correlation analysis of eccDNA load and the indicated clinical parameters. HRs adjusted for age, gender, BMI, and thyroid-related markers including FT3, FT4, TSH, TPOAb, and TgAb.


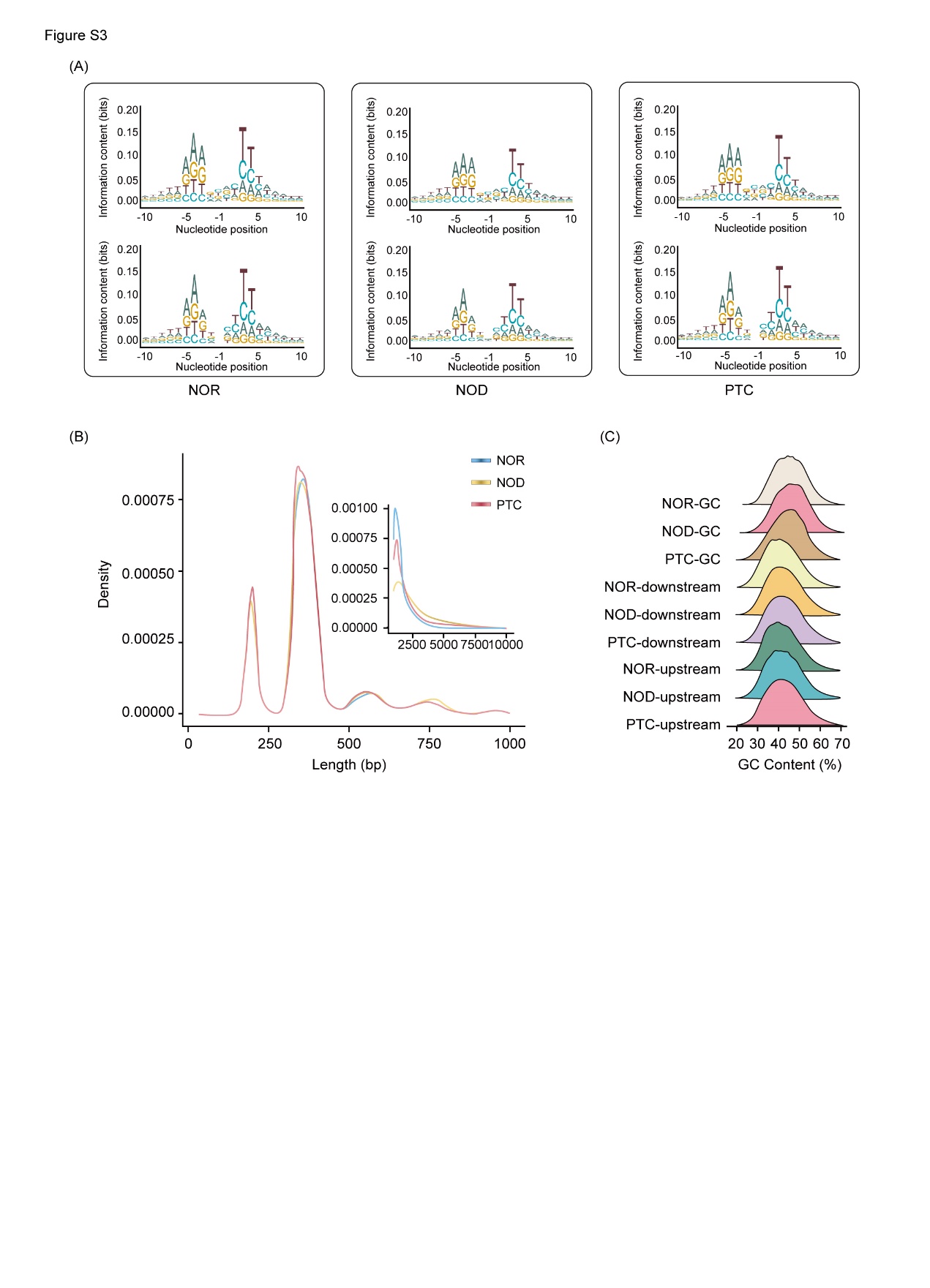


Figure S3. Baseline genomic information of eccDNA. (A) Motif patterns of eccDNA junction sites in each clinical group. (B) Size distribution of eccDNA in each clinical group. (C) GC content distribution of eccDNA and their up- and down-stream regions with equal length in each sample group.


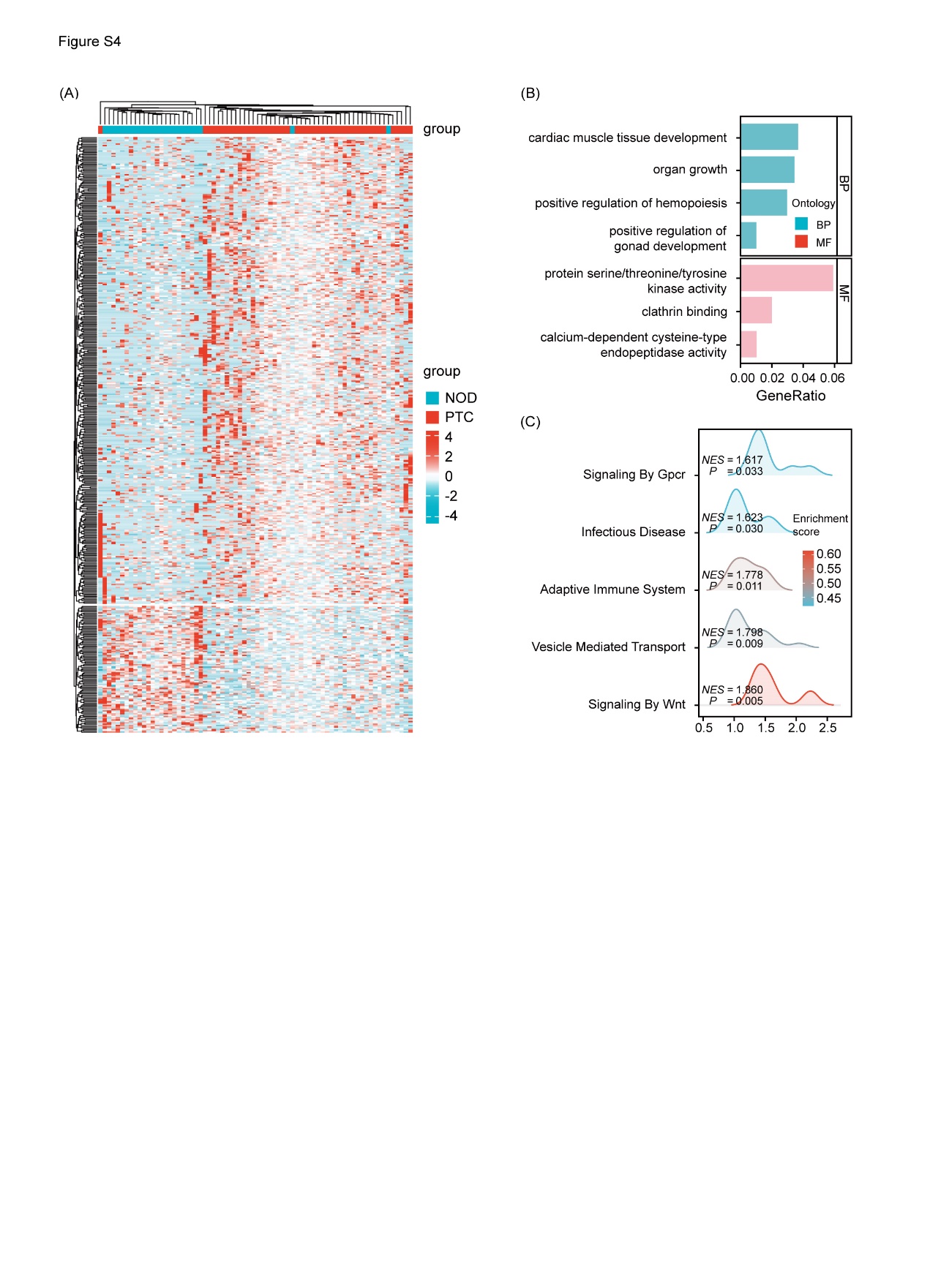


Figure S4. The differential of eccDNA-related genes in the different cohorts. (A) Heatmap showing the protein-coding genes with differential eccDNA abundance between the PTC and NOD groups. (B-C) GO and GSEA pathway analysis of differential eccGenes between the PTC and NOD groups.

**Supplementary Tables**

**Table S1. Baseline clinical features of enrolled samples.**

| characteristics | NOR | NOD | PTC | *P* value |
| --- | --- | --- | --- | --- |
| n | 13 | 25 | 47 |  |
| Sex, n (%) |  |  |  | 0.366 |
| Female | 7 (8.2%) | 19 (22.4%) | 33 (38.8%) |  |
| Male | 6 (7.1%) | 6 (7.1%) | 14 (16.5%) |  |
| Age, median (IQR)，y | 31.000 (27.000, 39.000) | 48 (40, 53) | 49 (33, 55.5) | 0.003 |
| Height, median (IQR) | 168.000 (166, 174) | 160 (156.75, 165.75) | 163 (160, 168) | 0.015 |
| Weight, median (IQR) | 66.000 (62, 69) | 64.75 (54.875, 73.25) | 67.5 (61, 75.5) | 0.341 |
| BMI, median (IQR) | 23.323 (21.799, 24.221) | 24 (21.148, 26.832) | 25.391 (23.234, 27.657) | 0.046 |
| **Thyroid Function** |  |  |  |  |
| FT3, mean ± SD | 4.4685 ± 0.32442 | 4.4146 ± 0.67149 | 4.445 ± 0.45469 | 0.949 |
| FT4, mean ± SD | 12.268 ± 1.1446 | 12.508 ± 1.1273 | 13.506 ± 2.2065 | 0.020 |
| TSH, median (IQR) | 1.9229 (1.3179, 2.3049) | 1.0412 (0.85333, 1.6997) | 2.25 (1.6, 3.26) | <0.001 |
| Hashimoto thyroiditis, n (%) |  |  |  | 0.086 |
| NO | 13 (15.3%) | 23 (27.1%) | 37 (43.5%) |  |
| Yes | 0 (0%) | 2 (2.4%) | 10 (11.8%) |  |
| **Liver Function** |  |  |  |  |
| TBIL (mol/L), median (IQR) | 11.44 (8.53, 14.51) | 14.18 (11.89, 17.58) | 13.6 (10.75, 16.4) | 0.087 |
| DBIL (μmol/L), median (IQR) | 2.17 (1.56, 2.72) | 2.8 (2.21, 3.47) | 3.8 (3.1, 4.65) | 0.000 |
| IBIL (μmol/L), median (IQR) | 9.1 (6.98, 11.79) | 11.47 (9.69, 14.36) | 9.7 (8, 12.6) | 0.030 |
| ALT (U/L), median (IQR) | 15.000 (12, 16) | 15 (10, 23) | 14 (10, 19) | 0.612 |
| AST (U/L), median (IQR) | 18.000 (17, 21) | 20 (17, 24) | 18 (16, 21) | 0.363 |
| **Serum Lipid** |  |  |  |  |
| TG (mmol/L), median (IQR) | 1.14 (0.88, 1.2) | 0.96 (0.81, 1.39) | 1.07 (0.87, 1.255) | 0.866 |
| TC (mmol/L), median (IQR) | 3.96 (3.75, 4.48) | 4.62 (4.385, 5.435) | 4.75 (4.275, 5.18) | 0.015 |
| HDL (mmol/L), median (IQR) | 1.21 (1.12, 1.3) | 1.36 (1.245, 1.595) | 1.16 (1.05, 1.375) | 0.054 |
| LDL (mmol/L), mean ± SD | 2.5369 ± 0.45591 | 2.91 ± 0.6766 | 2.9465 ± 0.67901 | 0.133 |
| **Tumor pathological features** |  |  |  |  |
| large tumor size (≥ 2cm) |  |  | 21(44.7%) |  |
| mulifocality, n (%) |  |  | 33 (70.2%) |  |
| lymphatic metastasis, n (%) |  |  | 36 (76.6%) |  |
| Extrathyroidal extension, n (%) |  |  | 43 (91.5%) |  |
| Pathologic stage, n (%) |  |  |  |  |
| I |  |  | 37 (78.7%) |  |
| II |  |  | 10 (21.3%) |  |
| **eccDNA counts** |  |  |  |  |
| epm, median (IQR) | 333.42 (230.12, 506.35) | 289.63 (257.43, 358.04) | 696.3 (490.47, 938.03) | <0.001 |
| eccNum, median (IQR) | 2.799e+04 (1.89e+04, 3.078e+04) | 1.569e+04 (1.077e+04, 1.891e+04) | 2.16e+04 (1.54e+04, 3.805e+04) | 0.009 |
| mappingCodingNum, median (IQR) | 1.467e+04 (9914, 1.598e+04) | 8356 (5758, 1.029e+04) | 1.128e+04 (8203, 2.024e+04) | 0.008 |
| mappingCodingRatio, mean ± SD | 52.437 ± 0.7195 | 52.62 ± 1.1381 | 53.476 ± 0.82264 | <0.001 |

**Table S2. Detailed information of the selected locations**

| Windows number | Chromosome | Local OR | Local *P* value | Gene name | Ensembl ID | Biotype |
| --- | --- | --- | --- | --- | --- | --- |
| 1 | 1 | 1.039981 | 0.000231 | SKI | ENSG00000157933 | protein_coding |
| 2 | 1 | 1.060186 | 0.000318 | PRDM16 | ENSG00000142611 | protein_coding |
| 3 | 1 | 0.872422 | 0.010583 | PRAMEF19 | ENSG00000204480 | protein_coding |
| 4 | 1 | 0.949254 | 0.036588 |  |  |  |
| 5 | 1 | 1.041004 | 6.81E-05 | ALDH4A1 | ENSG00000159423 | protein_coding |
| 6 | 1 | 1.044718 | 0.000712 | EPHB2 | ENSG00000133216 | protein_coding |
| 7 | 1 | 1.04438 | 0.000249 | SSBP3 | ENSG00000157216 | protein_coding |
|  |  |  |  |  | ENSG00000232245 | lncRNA |
| 8 | 1 | 1.056413 | 9.99E-05 | NFIA | ENSG00000162599 | protein_coding |
| 9 | 1 | 0.912761 | 0.029326 |  |  |  |
| 10 | 2 | 1.039205 | 0.001196 |  | ENSG00000237720 | lncRNA |
| 11 | 2 | 1.040715 | 0.000291 |  |  |  |
| 12 | 2 | 1.05868 | 0.000149 | SULT1C2P1 | ENSG00000237223 | transcribed_unprocessed_pseudogene |
|  |  |  |  | SULT1C2P1 | ENSG00000290794 | lncRNA |
| 13 | 2 | 0.931758 | 0.013036 | Y_RNA | ENSG00000200390 | misc_RNA |
| 14 | 2 | 0.942556 | 0.021298 |  |  |  |
| 15 | 2 | 0.931852 | 0.022139 | IRS1 | ENSG00000169047 | protein_coding |
| 16 | 2 | 1.054956 | 0.000132 |  |  |  |
| 17 | 3 | 1.051774 | 0.000202 |  |  |  |
| 18 | 3 | 1.06098 | 7.41E-05 | LINC01811 | ENSG00000226320 | lncRNA |
| 19 | 3 | 1.049674 | 0.000431 | ST3GAL6 | ENSG00000064225 | protein_coding |
| 20 | 3 | 1.063784 | 0.000168 | MGLL | ENSG00000074416 | protein_coding |
|  |  |  |  | KBTBD12 | ENSG00000187715 | protein_coding |
| 21 | 4 | 1.078204 | 0.000104 |  | ENSG00000246090 | lncRNA |
| 22 | 4 | 0.963823 | 0.069331 |  |  |  |
| 23 | 4 | 1.032176 | 0.043769 | TENM3-AS1 | ENSG00000177822 | lncRNA |
| 24 | 5 | 0.923196 | 0.036021 |  |  |  |
| 25 | 5 | 1.047981 | 0.000348 | F12 | ENSG00000131187 | protein_coding |
|  |  |  |  | PFN3 | ENSG00000196570 | protein_coding |
|  |  |  |  | GRK6 | ENSG00000198055 | protein_coding |
| 26 | 6 | 1.048198 | 0.000755 | SLC22A23 | ENSG00000137266 | protein_coding |
| 27 | 6 | 0.924564 | 0.036021 | NEDD9 | ENSG00000111859 | protein_coding |
| 28 | 6 | 1.061093 | 5.66E-05 | KIF13A | ENSG00000137177 | protein_coding |
| 29 | 6 | 1.052817 | 0.000681 | MIR548A1HG | ENSG00000283408 | lncRNA |
| 30 | 6 | 1.071285 | 0.000181 | TULP4 | ENSG00000130338 | protein_coding |
|  |  |  |  | SRP72P2 | ENSG00000188451 | processed_pseudogene |
| 31 | 7 | 0.972232 | 0.104673 | DNAAF5 | ENSG00000164818 | protein_coding |
| 32 | 7 | 1.046231 | 0.000402 | MAD1L1 | ENSG00000002822 | protein_coding |
|  |  |  |  |  | ENSG00000286192 | protein_coding |
| 33 | 7 | 0.91372 | 0.013698 | FBXL18 | ENSG00000155034 | protein_coding |
|  |  |  |  | MIR589 | ENSG00000207973 | miRNA |
| 34 | 7 | 1.06365 | 2.55E-05 |  |  |  |
| 35 | 7 | 0.940485 | 0.025157 | ZNF282 | ENSG00000170265 | protein_coding |
| 36 | 7 | 1.072648 | 0.000168 |  |  |  |
| 37 | 8 | 1.042402 | 0.000135 |  |  |  |
| 38 | 8 | 1.03422 | 0.003864 | MTRF1LP2 | ENSG00000224110 | processed_pseudogene |
|  |  |  |  |  | ENSG00000285108 | lncRNA |
| 39 | 9 | 1.066807 | 0.000168 | GOLM1 | ENSG00000135052 | protein_coding |
| 40 | 9 | 0.886074 | 0.03076 | PNPLA7 | ENSG00000130653 | protein_coding |
|  |  |  |  | DPH7 | ENSG00000148399 | protein_coding |
|  |  |  |  | MRPL41 | ENSG00000182154 | protein_coding |
| 41 | 10 | 1.06782 | 2.55E-05 |  |  |  |
| 42 | 10 | 1.041054 | 0.000402 | DRGX | ENSG00000165606 | protein_coding |
| 43 | 10 | 0.94617 | 0.022473 | LINC01163 | ENSG00000280953 | lncRNA |
| 44 | 11 | 1.041932 | 0.000288 |  |  |  |
| 45 | 11 | 1.043425 | 0.000568 | KCNQ1 | ENSG00000053918 | protein_coding |
| 46 | 11 | 1.053276 | 0.000149 | NUCB2 | ENSG00000070081 | protein_coding |
| 47 | 11 | 1.043392 | 9.63E-05 | MARK2 | ENSG00000072518 | protein_coding |
| 48 | 11 | 1.06195 | 0.000335 | PC | ENSG00000173599 | protein_coding |
|  |  |  |  | LRFN4 | ENSG00000173621 | protein_coding |
| 49 | 11 | 1.073898 | 0.000142 | ALG1L9P | ENSG00000248671 | lncRNA |
|  |  |  |  | ENPP7P8 | ENSG00000255319 | transcribed_unprocessed_pseudogene |
| 50 | 11 | 1.036899 | 0.000824 | DSCAML1 | ENSG00000177103 | protein_coding |
| 51 | 11 | 0.939787 | 0.04715 |  |  |  |
| 52 | 12 | 0.968879 | 0.05294 | GALNT8 | ENSG00000130035 | protein_coding |
|  |  |  |  |  | ENSG00000255639 | protein_coding |
| 53 | 12 | 0.89938 | 0.031708 | LINC02882 | ENSG00000251138 | lncRNA |
| 54 | 12 | 1.05257 | 0.000202 |  |  |  |
| 55 | 14 | 0.897699 | 0.011744 |  | ENSG00000258561 | lncRNA |
|  |  |  |  |  | ENSG00000287833 | lncRNA |
| 56 | 14 | 1.06707 | 9.49E-05 |  |  |  |
| 57 | 15 | 1.027771 | 0.001692 | SMAD3 | ENSG00000166949 | protein_coding |
| 58 | 15 | 1.01809 | 0.008686 | LINC02251 | ENSG00000258483 | lncRNA |
| 59 | 16 | 0.930535 | 0.036021 | MGRN1 | ENSG00000102858 | protein_coding |
| 60 | 16 | 0.92645 | 0.021983 | CLEC16A | ENSG00000038532 | protein_coding |
|  |  |  |  |  | ENSG00000274038 | lncRNA |
| 61 | 16 | 1.050428 | 7.41E-05 | SNX29 | ENSG00000048471 | protein_coding |
| 62 | 17 | 1.071249 | 4.88E-05 | ASIC2 | ENSG00000108684 | protein_coding |
| 63 | 17 | 1.058613 | 7.41E-05 | CACNG4 | ENSG00000075461 | protein_coding |
| 64 | 17 | 1.055418 | 0.000164 | SEPTIN9 | ENSG00000184640 | protein_coding |
| 65 | 18 | 1.057409 | 0.000156 |  | ENSG00000266924 | lncRNA |
| 66 | 19 | 1.053624 | 0.000163 | ZNF671 | ENSG00000083814 | protein_coding |
|  |  |  |  | ZNF551 | ENSG00000204519 | protein_coding |
|  |  |  |  |  | ENSG00000269026 | protein_coding |
| 67 | 20 | 1.050666 | 0.000233 |  |  |  |
| 68 | 20 | 0.901715 | 0.031708 |  |  |  |
| 69 | 20 | 1.050413 | 0.000295 | PRPF6 | ENSG00000101161 | protein_coding |
| 70 | 21 | 1.063967 | 0.000181 | LINC00163 | ENSG00000234880 | lncRNA |
| 71 | X | 0.907712 | 0.01753 | HMGN5 | ENSG00000198157 | protein_coding |

**Table S3. Outward PCR primers for eccDNA validation.**

| **sample** | **chromosome** | **Start** | **End** | **Elements** | **Discordant** | **Split** | **Size** | **Foward primer** | **Reverse primer** | **PCR length** |
| --- | --- | --- | --- | --- | --- | --- | --- | --- | --- | --- |
| PTC2 | chr17 | 48156378 | 48156726 | miRNA1203 | 3 | 25 | 348 | CGGGGAGGAAGGGAAGTTTAGT | CTCAAGCCACAGCAGGGTGTTT | 334 |
| PTC19 | chr17 | 48156324 | 48156528 | miRNA1203 | 0 | 27 | 204 | CCATTGGTTACGTATGAGGAGACCT | AGAGCCTGCGACTCGTAAAT | 198 |
| PTC26 | chr17 | 48156189 | 48156568 | miRNA1203 | 94 | 105 | 379 | GGAAGTAATTTACGAGTCGCAGGCT | GGAAAAGGTGTTTGAAAGGCCTGT | 334 |

**Table S4. LAMA primers of miRNA1203 eccDNA.**

| **Gene** | **sequence** | **Length** | **Forward Primer** | **Reverse primer** |
| --- | --- | --- | --- | --- |
| miRNA1203-A | ATTTACGAGTCGCAGGCTCTCAGGAGCCATTGGTTACGTATGAGGAGACCTGCACCAGCGCCACAATCTGGAGCCACTGAAGAGCGCTAAACACCCTGCTGTGGCTTGAGCTGCATCCTGGCTCCGGGGAGGAAGGGAAGTTTAGTCCCTCACCAGAAAAAGCTGTTCAGAAATATCTGAGTCCAAAAGGGACCGCCACGCTAAAACCACAGCCTCAATGCTGCCGAGTTTGCTTCTGTGAGATTAATTCTGTGAAATTAATTGGGACAAGATTGAAAAGGAAATTAAAATGCAGCTGAGTGAACAGGCCTTTCAAACACCTTTTCCCTGCTCTATTCAAACTTGAATTCTGCGGTGGTCGCCGGCATCGTGGGAAGTA | 379 | ATTTACGAGTCGCAGGCTCT | TACTTCCCACGATGCCGGCG |
| miRNA1203-B | GACCGCCACGCTAAAACCACAGCCTCAATGCTGCCGAGTTTGCTTCTGTGAGATTAATTCTGTGAAATTAATTGGGACAAGATTGAAAAGGAAATTAAAATGCAGCTGAGTGAACAGGCCTTTCAAACACCTTTTCCCTGCTCTATTCAAACTTGAATTCTGCGGTGGTCGCCGGCATCGTGGGAAGTAATTTACGAGTCGCAGGCTCTCAGGAGCCATTGGTTACGTATGAGGAGACCTGCACCAGCGCCACAATCTGGAGCCACTGAAGAGCGCTAAACACCCTGCTGTGGCTTGAGCTGCATCCTGGCTCCGGGGAGGAAGGGAAGTTTAGTCCCTCACCAGAAAAAGCTGTTCAGAAATATCTGAGTCCAAAAGG | 379 | GACCGCCACGCTAAAACCAC | CCTTTTGGACTCAGATATTTC |

**List of abbreviations**

| eccDNA | Extrachromosomal circular DNA |
| --- | --- |
| circle-seq | Circle sequencing |
| LOOCV | Leave-one-out cross-validation |
| PTC | Papillary thyroid carcinoma |
| NOD | Thyroid nodules |
| NOR | Healthy normal |
| AUC | Area under the curve |
| FNA | Fine needle aspiration |
| TI-RADS | Thyroid Imaging, Reporting, and Data system |
| NIPT | Non-invasive prenatal testing |
| TRIPOD | Transparent Reporting of a multivariable prediction model for Individual Prognosis or Diagnosis |
| RCA | Rolling circle amplification |
| NTC | Non-template control |
| LAMA | Ligase-assisted minicircle accumulation |
| ATCC | American type culture collection |
| FBS | Fetal bovine serum |
| DGE | Differential gene expression |
| SPF | Specific pathogen-free |
| dsDNA | Double-strand DNA |
| NGS | Next-generation sequencing |
| UTR | Untranslated regions |
| eccGenes | eccDNAs containing partial gene |
| GO | Gene Ontology |
| GSEA | Gene Set Enrichment Analysis |
